# Supplementary material for: Imitating by Generating: Deep Generative Models for Imitation of Interactive Tasks
Source: Front Robot AI. 2020 Apr 16;7:47. doi: 10.3389/frobt.2020.00047 (PMC7806025; doi:10.3389/frobt.2020.00047)
Supplement: Supplementary file 2 [file Data_Sheet_1.PDF]

Supplementary Material: Imitating by generating: deep generative models for imitation of interactive tasks

1 MODEL STRUCTURE

In this section we describe the components of our models in detail.

1.1 Latent variables

All latent variables $(\mathbf{z}_t^{s1}, \mathbf{z}_t^{s2}, \mathbf{z}_t^r, \mathbf{d}_t^s)$ are chosen to be independent and identically distributed Gaussian units with a trainable mean and variance. The prior is set to be standard normal distributed $p_{\theta_z}(\mathbf{z}_t) \sim \mathcal{N}(0, 1)$. The latent variables are trained with the reparameterization trick (Kingma and Welling, 2015; Rezende et al., 2014).

1.2 VAE embedding of human-human data

We embed $\mathbf{x}_{t:t+w}^s$, where $w = 40$. Thus the input layer has a dimension of 480: 40 times 4 (number of joints) times 3 (Cartesian dimensions). The VAE consists of dense layers with Relu non-linearities everywhere except the mean and variance layer of the latent space and the output layer, all of which are linear layers. The structure is as follows:

Input(480) - Relu(Dense(250)) - Relu(Dense(150)) - z[Dense(40), Dense(40)] - Relu(Dense(150)) - Relu(Dense(250)) - Dense(480).

Here z$[\mu, \sigma]$ represents the dimensions of the latent variables $\mathbf{z}_t^s$.

1.3 Task dynamics model of human-human data

We train two identical models for the two human partners.

Next to the current observation of the human joints $\mathbf{x}_t^s$ (dimension 4x3), we provide the network with a one-hot vector indicating which action is currently performed. We add an additional *not-active* action, which indicates those time steps after completion of the interaction. Thus, the one-hot vector $\mathbf{y}_t^s$ is of dimension 5. These two inputs are concatenated. The output of the network is an approximation of the latent variable $\mathbf{z}_t^s$. For all layers except those that indicate latent variables we use Tanh non-linearities. The recurrent cells are chosen to be Long short-term memory (LSTM) cells.

The structure of the network is as follows:

Concat(Input(12), Input(5)) - Tanh(LSTM(256)) - Tanh(LSTM(256)) - Tanh(LSTM(256)) - d[Dense(40), Dense(40)] - Tanh(Dense(40)) - Tanh(Dense(40)) - z[Dense(40), Dense(40)].

Here z$[\mu, \sigma]$ and d$[\mu, \sigma]$ represent the dimensions of the latent variables $\mathbf{z}_t^s$ and the dynamics $\mathbf{d}_t^s$ respectively.

1.4 Conditioned VAE embedding of human-robot data

Next to the last observation of the robot joints $\mathbf{x}_{t:t+w}^r$ (of dimension 7), we provide the network with the one-hot vector indicating which action is currently performed $\mathbf{y}_t^r$. Additionally, we provide the dynamics variable $\mathbf{d}_t$ (of dimension 40) extracted with the model described in Section 1.3. These three inputs are concatenated. The output of the network is an approximation of the latent variable $\mathbf{z}_t^r$. For all layers except

those that indicate latent variables we use Tanh non-linearities. The recurrent cells are chosen to be Long short-term memory (LSTM) cells.

The structure of the network is as follows:

Input(280) - Relu(Dense(250)) - Relu(Dense(100)) - Concat(Input(7), z[Dense(40), Dense(40)]) - Relu(Dense(100)) - Relu(Dense(250)) - Dense(280).

Here z[μ, σ] and represents the dimensions of the latent variables $\mathbf{z}_t^r$, which is the same as in Section 1.5.

1.5 VAE embedding of robot data

This model is required for the recurrent embedding described in Section 1.6. We embed $\mathbf{x}_{t:t+w}^r$, where $w = 40$. Thus the input layer has a dimension of 280: 40 times 7 (number of joints). The VAE consists of dense layers with Relu non-linearities everywhere except the mean and variance layer of the latent space and the output layer, all of which are linear layers. The structure is as follows:

Input(280) - Relu(Dense(250)) - Relu(Dense(150)) - z[Dense(7), Dense(7)] - Relu(Dense(150)) - Relu(Dense(250)) - Dense(280).

Here z[μ, σ] represents the dimensions of the latent variables $\mathbf{z}_t^r$.

1.6 Recurrent embedding of human-robot data

Next to the current observation of the robot joints $\mathbf{x}_t^r$ (of dimension 7), we provide the network with the one-hot vector indicating which action is currently performed $\mathbf{y}_t^r$. Additionally, we provide the dynamics variable $\mathbf{d}_t^s$ (of dimension 40) extracted with the model described in Section 1.3. These three inputs are concatenated. The output of the network is an approximation of the latent variable $\mathbf{z}_t^r$. For all layers except those that indicate latent variables we use Tanh non-linearities. The recurrent cells are chosen to be Long short-term memory (LSTM) cells.

The structure of the network is as follows:

Concat(Input(7), Input(5), Input(40)) - Tanh(LSTM(256)) - Tanh(LSTM(256)) - Tanh(LSTM(256)) - Tanh(Dense(40)) - Tanh(Dense(40)) - z[Dense(7), Dense(7)].

Here z[μ, σ] and represents the dimensions of the latent variables $\mathbf{z}_t^r$, which is the same as in Section 1.5.

REFERENCES

Kingma, D. P. and Welling, M. (2015). Auto-encoding variational bayes. *International Conference on Learning Representations (ICLR)*

Rezende, D. J., Mohamed, S., and Wierstra, D. (2014). Stochastic backpropagation and approximate inference in deep generative models. In *International Conference on Machine Learning*. 1278–1286
